# Altered Toll-like receptor expression and function in HPV-associated oropharyngeal carcinoma

**DOI:** 10.18632/oncotarget.18959

**Published:** 2017-07-04

**Authors:** Priscila Lie Tobouti, Robert Bolt, Raghu Radhakrishnan, Suzana Cantanhede Orsini Machado de Sousa, Keith D. Hunter

**Affiliations:** ^1^ Oral Pathology Department, School of Dentistry, University of São Paulo, São Paulo, Brazil; ^2^ Unit of Oral and Maxillofacial Pathology, School of Clinical Dentistry, University of Sheffield, Sheffield, UK; ^3^ Department of Oral Pathology, Manipal College of Dental Sciences, Manipal University, Manipal, India

**Keywords:** HPV, oropharyngeal squamous cell carcinoma, toll-like receptor, interleukin-6, interleukin-8

## Abstract

Toll-like receptors (TLRs) have been widely investigated due to their importance in the inflammatory response and possible links to tumor promotion/regression and prognosis. In cancers with an infective etiology, such as human papillomavirus (HPV)-associated Oropharyngeal Squamous Cell Carcinoma (OPSCC), TLR responses may be activated and play a role in tumorigenesis. Our aim was to assess the expression of all TLRs in OPSCC cell lines (both HPV^+^ and HPV^–^) by qPCR, Western Blot and flow cytometry and assess their response to TLR ligands lipopolysaccharide (LPS), LPS ultra-pure (LPS-UP) and peptidoglycan (PGN) by analyzing IL-8 and IL-6 production. We also immunostained 61 OPSCC tissue samples with anti-TLR4. Results showed lower TLR1 and TLR6 mRNA expression and higher TLR9 protein expression in HPV^+^ when compared to HPV^–^OPSCC cells. TLR4 expression did not vary by HPV status in OPSCC cells, but TLR4 expression was significantly lower in HPV^+^OPSCC tissues. After stimulation with PGN, only one cell line (HPV+) did not secrete IL-6 or IL-8. Furthermore, HPV^+^OPSCC lines showed no IL-6 or IL-8 production on treatment with LPS/LPS-UP. The data suggest changes in TLR4 signaling in HPV^+^OPSCC, since we have shown lower tissue expression of TLR4 and no pro-inflammatory response after stimulation with LPS and LPS-UP. Also, it suggests that OPSCC may respond to HPV infection by increased expression of TLR9. This study demonstrates differences in expression and function of TLRs in OPSCC, which are dependent on HPV status, and may indicate subversion of the innate immune response by HPV infection.

## INTRODUCTION

The innate immune response detects pathogenic microorganisms through a number of mechanisms, including recognition of pathogen-associated molecular patterns (PAMP) by pattern-recognition receptors (PRRs), which include the Toll-like receptors (TLR) [1]. Most epithelial cells express TLRs, as well as relevant co-receptors and adapter molecules, such as MyD88 and CD14 [2]. The near-ubiquitous nature of TLR expression within normal epithelia relates to its important barrier function against invading microorganisms; TLR activity is essential for an effective host response to be mounted [3]. In addition to this central role in protection against infection, TLRs have roles in maintaining tissue homeostasis through the regulation of inflammatory and reparative responses to tissue injury [4].

Over 150 years ago, Virchow demonstrated a connection between inflammation and cancer by observing leucocytes within tumor tissue. Today, there is a wide consensus on the importance of the inflammatory response in tumor propagation and as a risk factor for carcinogenesis [5]. Inflammation can exert anti-apoptotic effects, induce oxidative DNA damage and promote a tissue reparative response. Concomitantly, the adaptive immune response is also important in tumor progression [6]. The link between the immune system and cancer progression has led several groups to assess the role of receptors capable of activating signaling pathways for the recruitment of inflammatory cells, among which are included the TLRs.

Although the TLR system has the capacity to respond to a range of microbial factors, individual TLRs demonstrate relatively high ligand specificity. TLR1, TLR2 and TLR6 primarily recognize peptidoglycan (PGN), lipoteichoic acid and zymogen; TLR3, double stranded RNA; TLR4, lipopolysaccharides (LPS) of Gram-negative bacteria; TLR5, bacterial flagellum; TLR7 and TLR8, single-stranded RNA; TLR9, bacterial and viral unmethylated CpG DNA [1, 4]. TLR10 is the only receptor without a specific known ligand [7].

Changes in the expression of TLRs or their signaling pathways may lead to progression or regression of the tumor, depending on cancer type [8]. Many studies have focused on the role of TLRs in cancer immunotherapy [8–10]; although results vary depending on cancer type and location. Individual studies are therefore necessary in order to assess the role of TLRs in a given cancer type, as the effects of TLR activation appear to be context-dependent and therefore cannot be predicted from data relating to other tumors.

Patients with HPV^+^ OPSCC have a better prognosis than those with HPV-negative disease [11, 12], but so far there are few studies correlating OPSCC HPV status to immune response [13]. In addition, studies in cervical cancer have shown changes in the innate immune response linked to the expression of TLRs [14, 15]. In oropharyngeal cancer, only two studies have been published that have evaluated Toll-like receptors expression [13, 16].

Our overall aim was to describe the patterns of expression of all TLRs in HPV^+^ and HPV^–^ OPSCC, and determine the function of selected receptors after stimulation with LPS and PGN, in order to determine the extent of alterations in TLR expression and function in OPSCC.

## RESULTS

### TLR1 and TLR6

**Figure 1:**
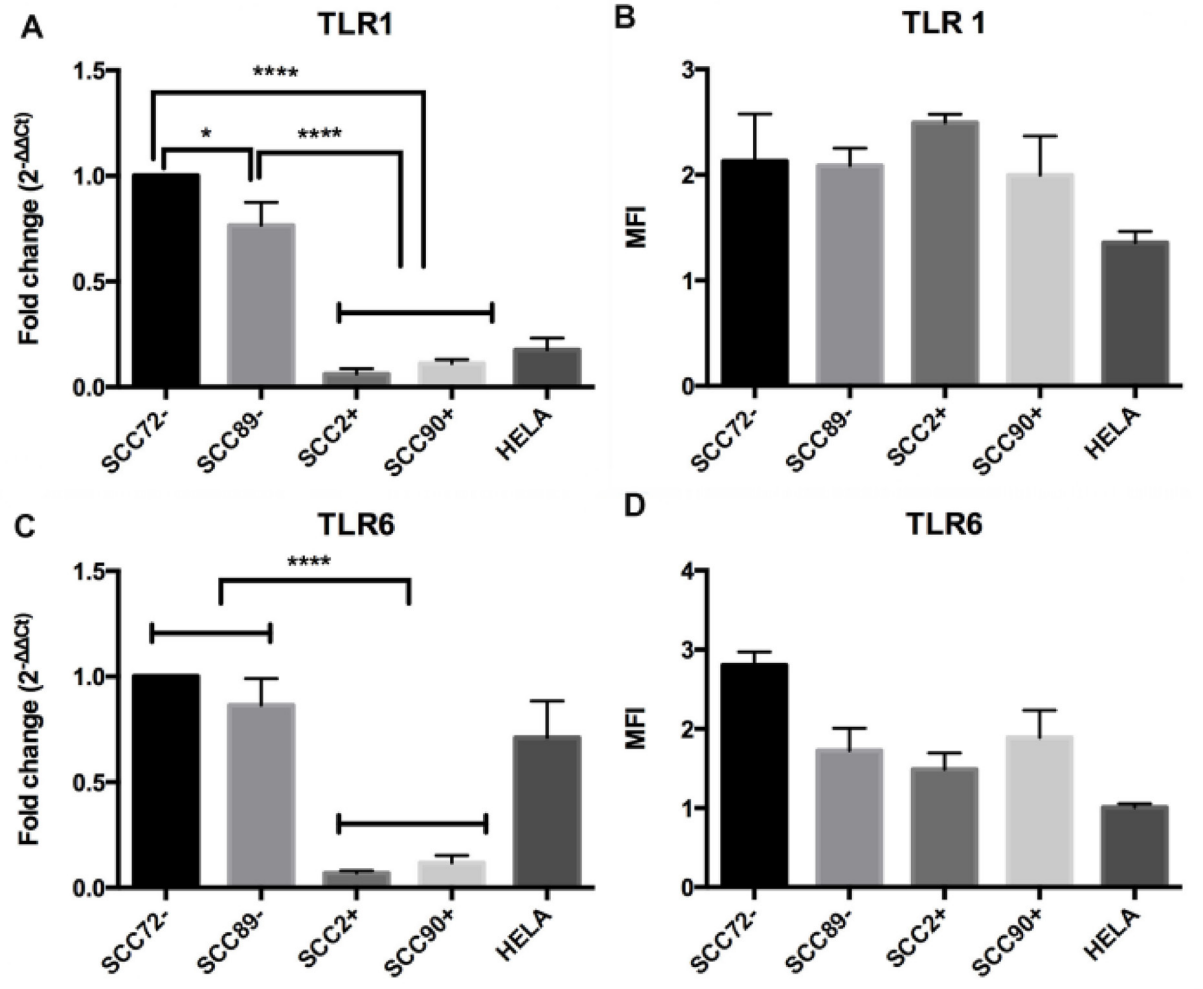
Expression of TLR1 and TLR6 in HPV^–^ (SCC72 and SCC89), HPV16^+^ (SCC2 and SCC90) and HPV18^+^ (Hela) (**A**) TLR1 mRNA expression relative to SCC72, by qPCR: significant higher levels of gene expression in SCC72 and SCC89 cell lines (HPV^-^) when compared to SCC2, SCC90 (HPV^+^); (**B**) TLR1 protein expression: Median Fluorescence intensity (MFI), analyzed by flow cytometry, did not show significant differences between cell lines (Median ± SD). (**C**) TLR6 mRNA expression relative to SCC72: significant higher levels of gene expression in SCC72 and SCC89 cell lines (HPV^-^) when compared to SCC2, SCC90 (HPV^+^); (**D**) TLR6 protein expression: did not show significant differences between cell lines (Median ± SD). (^*^ *p <* 0.05; ^****^p < 0.0001; Median ± SEM).

The same pattern of mRNA expression was seen for TLR1 (Figure 1A and 1B) and TLR6 (Figure 1C and 1D). Under unstimulated conditions, there were significantly higher levels of TLR1 and TLR6 gene expression in cell lines SCC72 and SCC89 (HPV^-^) when compared to lines SCC2 and SCC90 (HPV^+^), *p <* 0.0001. Significant differences of TLR1 gene expression were also noted between HPV^–^ cell lines (SCC72 and SCC89), *p <* 0.05 (Figure 1A). Despite differences in mRNA expression, there were no significant differences in TLR1 or TLR6 protein expression when comparing HPV^+^ and HPV^–^ cell lines, as measured by flow cytometry (Figure 1B).

### TLR2

**Figure 2:**
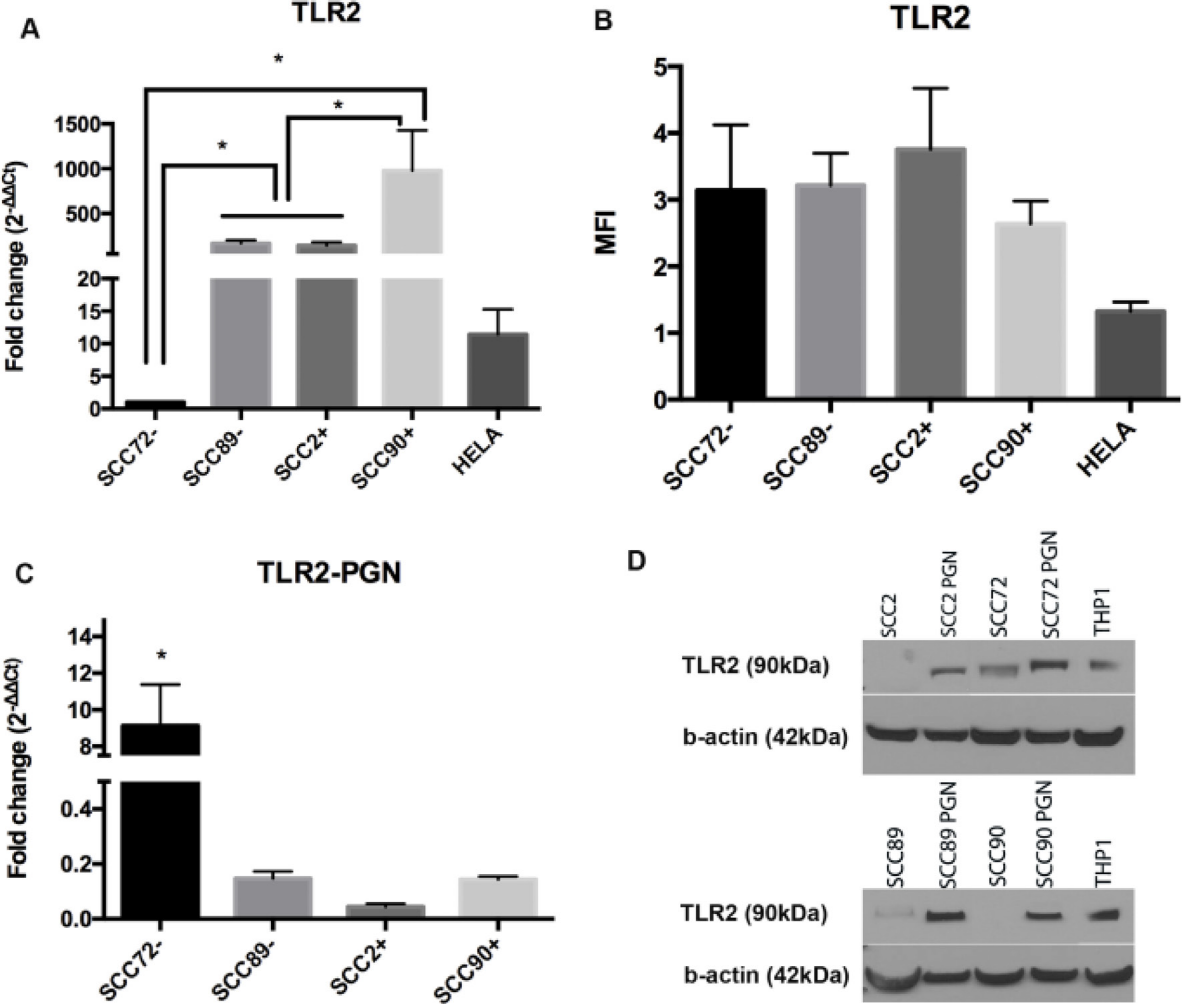
Expression of TLR2 in HPV^–^ (SCC72 and SCC89), HPV16^+^ (SCC2 and SCC90) and HPV18^+^ (Hela) (**A**) TLR2 gene expression relative to SCC72, by qPCR, shows significant difference between SCC72 and the other cell lines and between SCC89 and SCC2 compared to SCC90 (Median ± SEM, *p <* 0.01); (**B**) Median Fluorescence intensity (MFI), analyzed by flow cytometry: no significant difference between cell lines (Median ± SD), (**C**) TLR2 gene expression relative to SCC72 after PGN stimulus: SCC72 showed a significant difference between control and stimulated cells (Median ± SEM, *p <* 0.001); (**D**) Comparison of TLR2 expression between control (not stimulated) and cells stimulated with PGN: after stimulus all the cell lines expressed TLR2 (cropped gel). THP1 was used as control.

There was significantly lower expression of TLR2 mRNA in SCC72 compared to the other cell lines (SCC89, SCC2 and SCC90), *p <* 0.01 (Figure 2A). Significant differences were also noted between SCC90 and all other cell lines. Again, despite findings at the mRNA level, there were no differences in TLR2 protein expression when analyzed by flow cytometry (Figure 2B).

### TLR2 expression after stimulation with PGN

PGN stimulation of cell lines led to no significant change in TLR2 mRNA expression, with the exception of SCC72, *p <* 0.001 (Figure 2C), but it was possible to demonstrate higher expression of TLR2 protein in response to PGN in all cell lines (Figure 2D).

### Pro-inflammatory (IL-6 and IL-8) response after PGN stimulation

**Figure 3:**
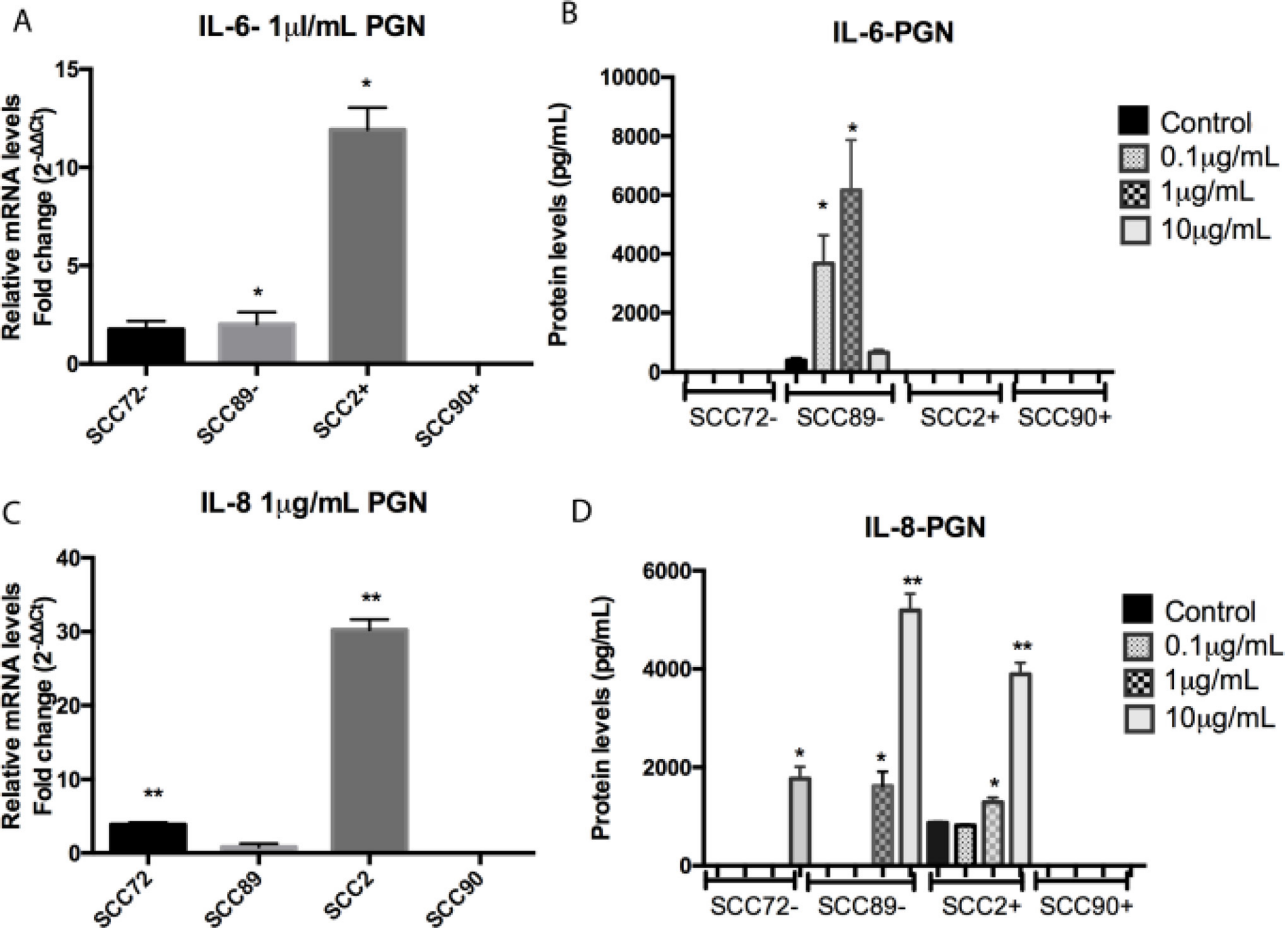
Comparison of IL-6 and IL-8 expression between non stimulated cells and cells stimulated with peptidoglycan (PGN) (**A**) IL-6 mRNA expression, relative to SCC72, was significantly increased in SCC89 and SCC2 after stimulation with 1 µg/mL (^*^p < 0.05; mean ± SEM); (**B**) Protein expression of IL-6 was increased in SCC89 after stimulation with 0.1 and 1 µg/mL PGN (^*^p < 0.0001; mean ± SD); (**C**) IL-8 mRNA expression, relative to SCC72, was significantly increased in SCC89 and SCC2 after stimulation with 1 µg/mL of PGN (^**^p < 0.001; mean ± SEM); (**D**) Protein expression of IL-8 was increased in SCC72 after stimulation with 10 µg/mL, and SCC89 and SCC2 after stimulation with 1 and 10 µg/mL of PGN (^*^p < 0.01, ^**^p < 0.0001; mean ± SD).

PGN stimulation of the cell line SCC72 (HPV^-^) did not demonstrate significant change in IL-6 expression (Figure 3A and 3B). However, PGN treatment led to higher expression of IL-8 mRNA after stimulation with 1 µg/mL PGN (Figure 3C) and higher protein expression after 10 µg/mL PGN, *p <* 0.001 (Figure 3C).

After 1 µg/mL PGN stimulation, SCC89 (HPV^-^) demonstrated an increase in IL-6 mRNA expression (Figure 3A), as well as significantly higher cytokine secretion after stimulation with 0.1 µg/mL and 1 µg/mL PGN (Figure 3B), *p <* 0.0001, when compared to unstimulated control. In the same way, SCC89 demonstrated significantly higher IL-8 protein secretion after stimulation with 1 µg/mL and 10 µg/mL PGN (Figure 3D), *p <* 0.01. Despite the changes seen at the protein level, there was no significant change in IL-8 mRNA expression under stimulated conditions (Figure 3C).

There was no increase in IL-6 secretion in both HPV^+^ cell lines. Although SCC2 (HPV^+^) demonstrated higher levels of IL-6 mRNA in response to PGN (Figure 3A), no change was noted at the protein level (Figure 3B). Similarly, SCC90 (HPV^+^) did not express IL-6 or IL-8 at either the mRNA or protein level under either basal or stimulated conditions, *p <* 0.001 (Figure 3B and 3D).

SCC90 (HPV^+^) was the only OPSCC cell line tested which did not show any pro-inflammatory response to PGN stimulation; cell line SCC2 did exhibit increased IL-8 mRNA and protein expression after stimulation with 1 µg/mL PGN (Figure 3C) and also protein expression after 1 µg/mL and 10 µg/mL PGN stimulation, *p <* 0.01 (Figure 3D).

## TLR4

### TLR4 mRNA expression, but not protein expression, varies by HPV status *in vitro*

**Figure 4:**
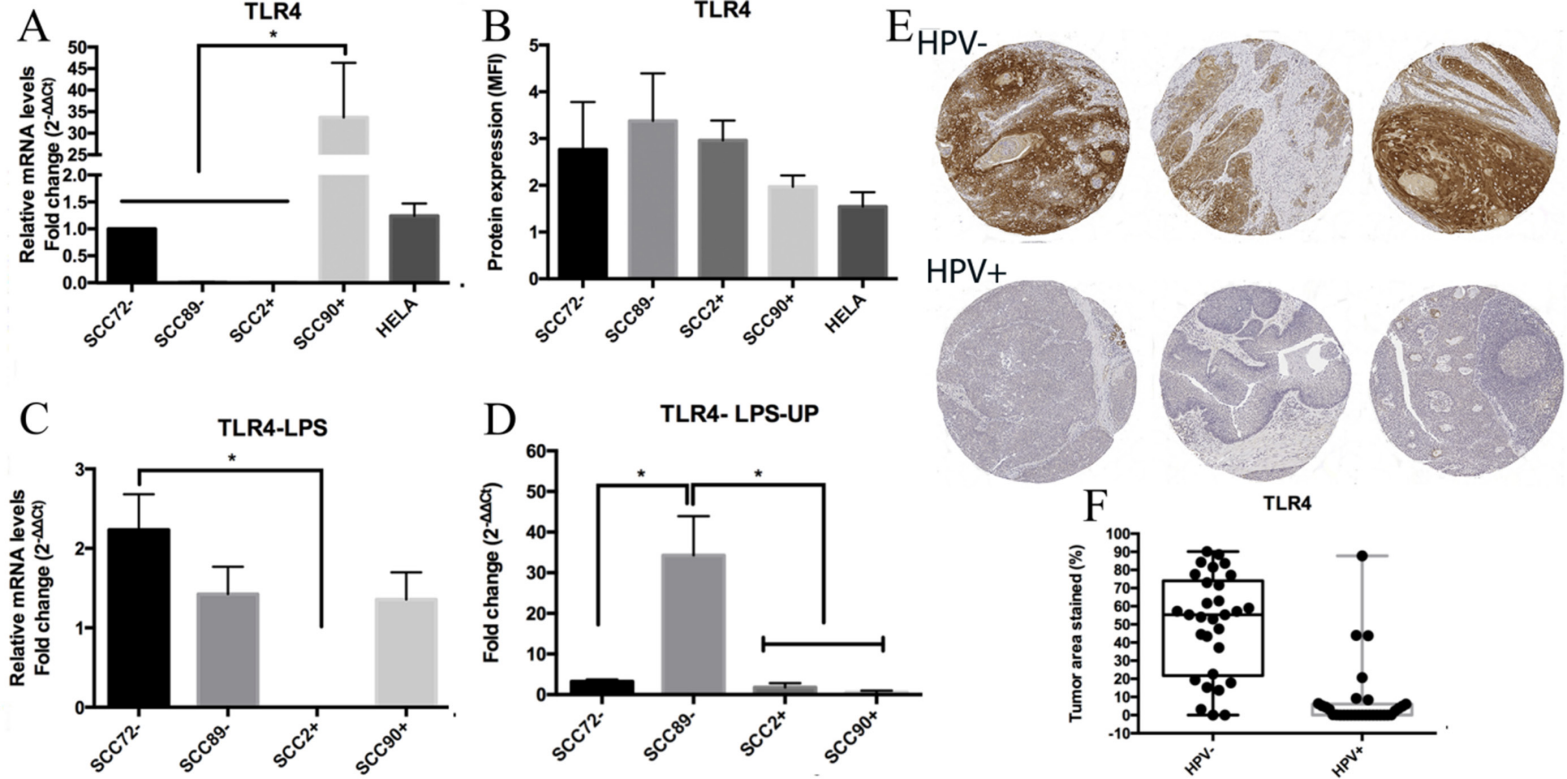
TLR4 expression (**A**) TLR4 gene expression, relative to SCC72, showed significant difference between SCC90 and the other cell lines; no significant differences between both HPVs^-^ and both HPVs^+^, by qPCR (target gene normalized to U6) (^**^p < 0.0001; Mean ± SEM); (**B**) Median Fluorescence intensity (MFI) showed no significant difference between cell lines, by flow cytometry (Mean ± SD); (**C**) TLR4 expression, relative to SCC72, between non stimulated cells and cells stimulated with LPS: significant higher difference between SCC72 and SCC2 (^*^p < 0.01; mean ± SEM); (**D**) TLR4 expression between non stimulated cells and cells stimulated with LPS ultra pure (LPS-UP): significant difference between SCC89 and the other cell lines (^*^p < 0.01; mean ± SEM); (**E**) anti-TLR4 stain in HPV^–^ tumors and HPV^+^ tumors: Lower expression of TLR4 in HPV-associated OPSCC; (**F**) Box and whisker plot of TLR4 stain in HPV^–^ and HPV^+^ OPSCC: significantly higher stain in HPV^–^ tumors (*p <* 0.0001).

The relative TLR4 gene expression results showed significant differences between SCC90 and the other OPSCC cell lines (SCC72, SCC89 and SCC2), *p <* 0.0001 (Figure 4A). No significant difference between cell lines was seen in the TLR4 protein expression (Figure 4B).

### TLR4 expression after stimulation with LPS and LPS-UP

After stimulation with the TLR4 agonist LPS, SCC72 showed significantly higher TLR4 expression in comparison to SCC2 (*p <* 0.01), however there was no consistent difference between HPV^+^ and HPV^–^ groups (Figure 4C). After stimulation with LPS-UP, SCC89 showed significantly higher mRNA expression of TLR4 in comparison to the other OPSCC cell lines, *p <* 0.01 (Figure 4D).

### Lower expression of TLR4 in HPV^+^ OPSCC tissues

Immunohistochemical staining was restricted to cell membrane and/or the cytoplasm. No nuclear staining was identified in any of the samples. Significantly lower expression of TLR4 was observed in HPV^+^ tumors when compared to HPV^–^ tumors, *p <* 0.0001 (Figure 4E and 4F).

### HPV-associated OPSCC shows no consistent pro-inflammatory (IL-6 or IL-8) response after stimulation with LPS and LPS-UP

**Figure 5:**
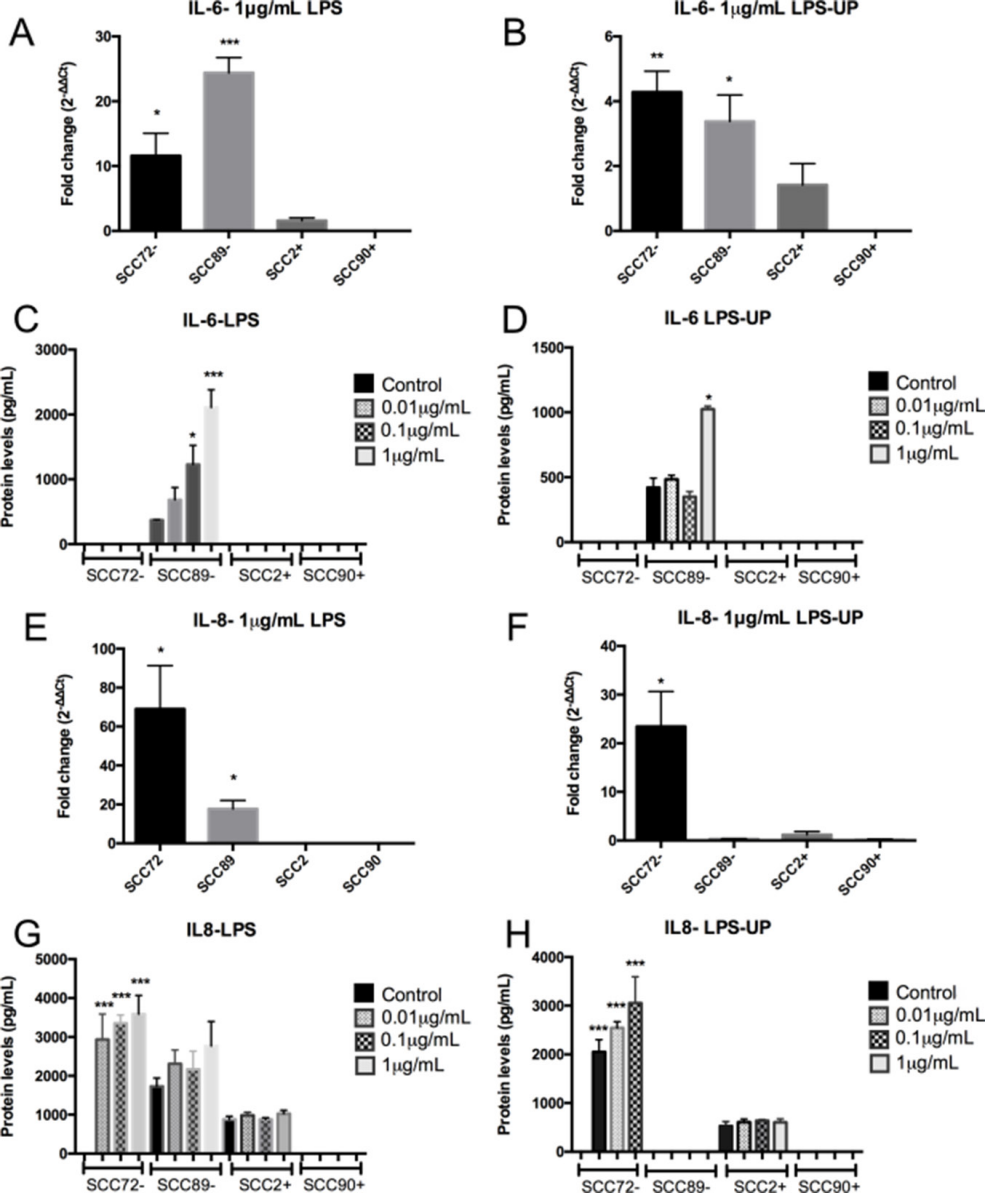
HPV-associated OPSCC shows no pro-inflammatory IL-6 or IL-8 response after stimulation with LPS and LPS ultra pure (LPS-UP) (**A**) and (**B**) IL-6 mRNA expression, relative to the untreated, show increase in HPV^–^ cell lines (SCC72 and SCC89) after stimulation with 1 µg/mL of LPS. SCC2 and SCC90 (HPV^+^) did not show increase of IL-6 expression after stimulation (^*^p < 0.05, ^**^p < 0.01; ^***^p < 0.001; Mean ± SEM); (**C**) Increase of protein levels of IL-6 in SCC89 after stimulation with 0.1 µg/mL and 1 µg/mL of LPS (^**^p < 0.01; ^***^p < 0.001; Mean ± SD); (**D**) SCC72 showed significant increase of IL-6 after stimulation with 1 µg/mL of LPS-UP (^*^p < 0.01; Mean ± SD); (**E**) Only HPV^+^ cell lines showed no IL-8 gene expression, relative to the untreated, after stimulation with LPS (^*^p < 0.05; Mean ± SD); (**F**) Increase of IL-8 in SCC72 after stimulation with 1 µg/mL of LPS-UP, relative to the untreated (^*^p = 0.0353; Mean ± SD); (**G** and **H**) Increase of IL-8 in SCC72 after stimulation with 0.01, 0.1 and 1 µg/mL of LPS (^***^p < 0.0001; Mean ± SD).

There was no change in IL-6 (Figure 5A–5D) or IL-8 gene expression or protein secretion (Figure 5E–5H), after LPS and LPS-UP stimulation in SCC2 and SCC90. However, the HPV^–^ cell lines (SCC72 and SCC89) increased production of IL-6 or IL-8 (Figure 5).

Stimulation of SCC72 (HPV^–^) with either LPS or LPS-UP, led to both increased IL-8 mRNA expression (Figure 5E and 5F) and protein secretion (Figure 5G and 5H) over all concentrations tested. Furthermore, IL-6 mRNA expression was increased after stimulation with 1 µg/mL LPS or LPS-UP (Figure 5A and 5B), in comparison to unstimulated control, however increased protein secretion was not detected (Figure 5C and 5D).

In SCC89 (HPV^–^), after stimulation with 1 µg/mL of LPS or LPS-UP, higher gene expression of IL-6 was detected compared to unstimulated control (Figure 5A and 5B). This result mirrors findings changes in protein expression, where elevated concentrations of IL-6 were detected by ELISA after stimulation with 0.1 µg/mL and 1 µg/mL LPS (Figure 5C) or 1 µg/mL LPS-UP (Figure 5D). LPS treatment also resulted in higher IL-8 gene expression in SCC89 (Figure 5E), however no significant difference was observed in IL-8 protein secretion.

### TLR9

**Figure 6:**
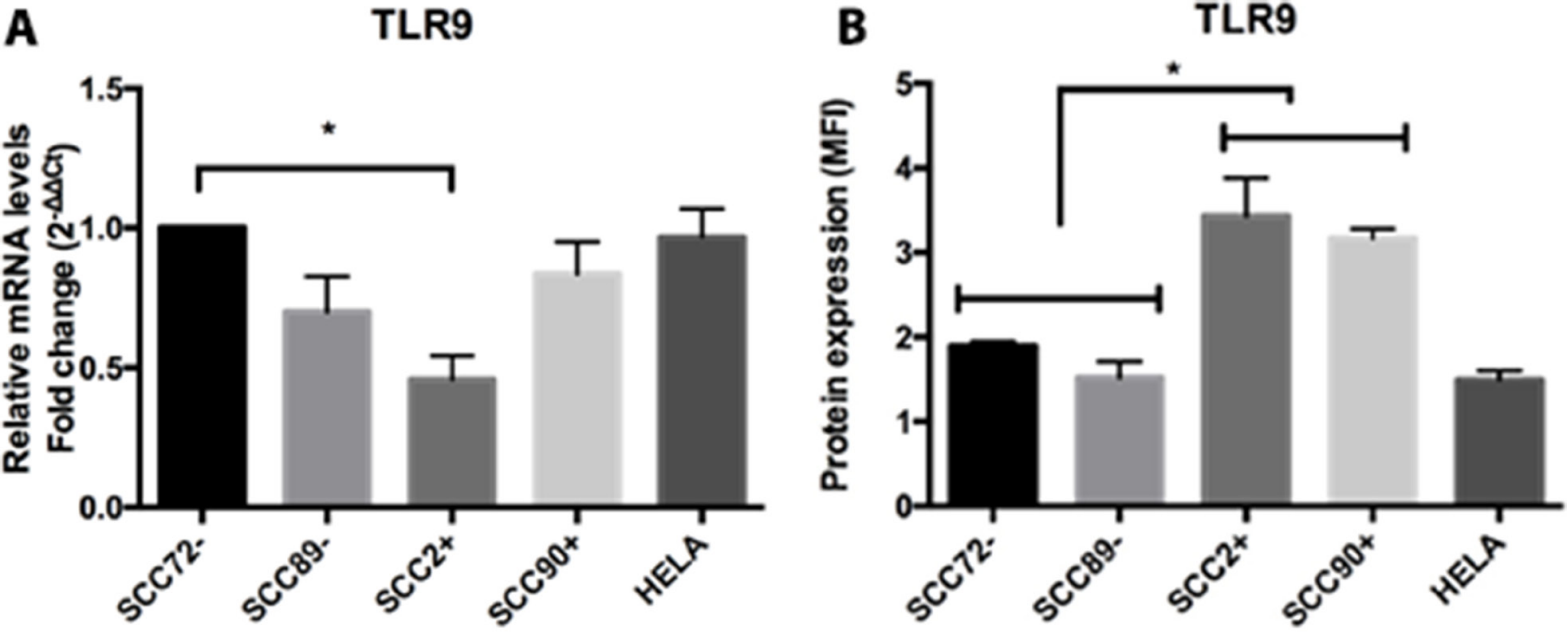
Expression of TLR9 in HPV^–^ (SCC72 and SCC89), HPV16^+^ (SCC2 and SCC90) and HPV18^+^ (Hela) (**A**) TLR9 gene expression, relative to SCC72, by qPCR, shows only significant difference between SCC72 and SCC2 (^*^p < 0.05); (**B**) Median Fluorescence intensity (MFI), analyzed by flow cytometry, show significant higher expression in HPV^+^ cell lines (SCC2 and SCC90) compared to HPV^–^ cell lines (SCC72 and SCC89) (^*^p < 0.01; Mean ± SD).

There were no significant differences in overall TLR9 mRNA expression on comparison of HPV^+^ and HPV^–^ cell lines, but the expression of TLR9 mRNA was higher in SCC72 than SCC2 (Figure 6A), *p <* 0.05. Despite no difference at mRNA expression, higher TLR9 protein expression was seen in HPV^+^ cell lines (SCC2 and SCC90) compared to HPV^–^ cell lines (SCC89 and SCC90), as measured by flow cytometry (Figure 6B).

### TLR3, 5, 7, 8 and 10

No evidence of significant differences in TLR 3, 5, 7, 8 or 10 gene expression between HPV^+^ and HPV^–^ lineages were noted. However, TLR3, without stimulation, demonstrated a significant difference in relative gene expression between SCC89 and the other cell lines studied (SCC72, SCC2 and SCC90) (Supplementary Figure 2), also a higher gene expression of TLR5 in SCC72 compared to SCC89, SCC2 and SCC90, as well as between SCC90 and both SCC2 and SCC89 was observed (Supplementary Figure 2). TLR7 did not show significant differences between HPV^+^ and HPV^–^ and the only cell line which expressed the gene was SCC89. There was no gene expression of TLR8 and TLR10 of the OPSCC cell lines used in this study.

## DISCUSSION

HPV-associated OPSCC shows clinical, pathological and biologically distinct features, due to viral activity within tumour tissue [17, 18]. HPV integrates into the host genome and expresses oncoproteins E6 and E7, which inactivate p53 and retinoblastoma (Rb), respectively and dysregulates the cell cycle [19]. HPV also appears to modify the expression and functionality of TLRs in cervical cancer [14, 15, 20, 21]. Mammalian TLRs help clear microbial infection [22], and have been correlated with both progression and regression of malignant tumors [8].

In this study, where we evaluated TLR 1-10 mRNA expression by qPCR, we observed that only TLR1 and TLR6 mRNA are more expressed in HPV^–^ when compared to HPV^+^ cell lines. Despite this finding, there were no significant differences in membranous TLR1 and TLR6 expression on comparing HPV^+^ and HPV^–^ OPSCC cells. De Carlo reported a similar decrease in TLR1 gene expression in HPV^+^ cervical cancers [23]. In an animal model, synthetic bacterial lipoprotein (TLR1 agonist) induced tumor regression by increasing cytotoxic T lymphocyte function [24]. The concept of a universal role for TLR1 in tumor regression is however undermined by data suggesting that it may promote esophageal adenocarcinoma [8].

The lack of any difference in TLR2 expression at either gene or protein level, between HPV^+^ and HPV^–^ cell lines, led us to assess the functionality of TLR2 after stimulation with PGN (a TLR2 agonist). PGN from *S. aureus* is an activator of TLR2 [25] and increases TLR2 expression after treatment, as observed in our work and similarly reported in the literature [25]. HPV^–^ lines SCC72 and SCC89 expressed IL-8 and IL-6, respectively, after stimulation with PGN. The HPV^+^ cell line SCC2 also expressed IL-8 after stimulation. This suggests functional TLR2 in HPV^–^ and HPV^+^ cells. However, intracellular receptors such as NOD (nucleotide-binding oligomerization domain) 1 and NOD2 can recognized peptides derived from the degradation of PGN. If NOD1 or NOD2 are stimulated, it could induce secretion of interleukins [26, 27], thus more studies are required to verify the true involvement of. TLR2 in HPV-associated OPSCC.

TLR4, a cell membrane receptor, has also been implicated in tumor promotion [8]. Immunohistochemical analysis of tumor specimens confirmed significantly lower expression of TLR4 protein in HPV^+^ tumors compared to HPV^–^ tumors *ex-vivo*. This finding is consistent with previous work assessing HPV- associated HNC [16]. Our *in vitro* findings suggested no clear relationship between TLR4 gene expression in HPV^+^ versus HPV^–^ cell lines; all cell lines expressed TLR4 when assessed by flow cytometry. Despite conflicting with *ex-vivo* findings, our *in vitro* work is also consistent with data published by Jouhi et al [13].

Unlike the upregulated release of inflammatory mediators observed in response to PGN, stimulation with LPS and LPS-UP resulted in no change in IL-6 or IL-8 expression in HPV^+^ cell lines, although we noted variable increased secretion of IL-6 and IL-8 in HPV^–^ cell lines. The lack of response in HPV^+^ lines may suggest changes in the TLR4 signaling pathway as a consequence of viral infection. In cervical cancer, the reported changes in expression of TLR4 are a source of controversy and effects seen may be related to the HPV type and different material and methods used; the cervical carcinoma lines SiHa (HPV16^+^) show higher expression of TLR4 than HeLa (HPV18^+^), moreover SiHa, but not HeLa display resistance to apoptosis following treatment with LPS, via TLR4 [21]. In cervical carcinoma, some authors have demonstrated high TLR4 expression [21, 28], whilst others demonstrated low TLR4 expression, linked to histological grade [14]. However, not all studies reported the HPV subtype involved. Interestingly, lack of or block of TLR4, IL-6 and IL-8 in many tumors has been related to better treatment response and prognosis [8, 29–33], responses also seen in HPV-associated OPSCC [11, 12, 34].

Many carcinomas have been found to express high levels of IL-6 and/or IL-8, suggesting an important role of these cytokines in the tumor microenvironment [35, 36]. IL-6 is implicated with a number of defense mechanisms, in normal tissue, as well as control of growth and differentiation in various malignancies [36]. Overexpression of IL-6 is also associated with tumor progression by inhibition of apoptosis [36], stimulation angiogenesis [37, 38] and reinforcing tumor drug resistance [39]. Similarly, IL-8 promotes a number of responses implicated with tumor progression, including angiogenesis, increased tumor proliferation & survival, and neutrophil chemo-attraction [35]. Moreover, IL-8 has been correlated to metastasis, as seen in orthotopic, xenograft and nude mouse models [40, 41]. In this context, increased levels of IL-8 confer resistance to chemotherapeutic agents in tumor cells [33] and its inhibition correlates to better tumor response to therapy [35]. Given the extensive tumor-promoting functions reported for both IL-6 and IL-8, dysfunction of the TLR4 signaling pathway which thereafter leads to reduced secretion of IL-6 and IL-8, could contribute to a better tumor prognosis, as seen in patients with HPV^+^ OPSCC.

Polymorphisms of TLRs have been associated with many diseases, and may be a risk factor linked to cervical cancer [42]. In addition to TLR4 polymorphisms, dysregulation of adapter molecules offers a further mechanism through which TLR4 function may be disrupted. For example, HeLa cells express TLR4 but not MD2, which is required for the activation of TLR4 in response to PAMPs [43]. Molecules such as CD14 and MD2 are also essential for LPS recognition and activation of the signaling pathway [44].

TLR9 has been the focus of numerous studies into cervical carcinoma, due to this receptor acting as a method of HPV recognition [45]. A decrease in host epithelial TLR9 expression may offer an opportunity for HPV to more effectively evade the immune system, thereby allowing viral persistence within infected cells. This deficiency may be further compounded by the TLR9-inhibiting effects of the E7 oncogene, once viral infection in established. The HPV16 virion has also been found to inhibit the transcriptional activity of TLR9 [45]. Despite the potential for downregulated TLR9 to be associated with viral infection, our findings were to the contrary, with higher expression observed in HPV^+^ lines and lower expression in HPV^–^ lines.

Interestingly, Parroche et al., 2016, demonstrated downregulation of TLR9 in HNC (HPV negative) and showed that TLR9 induced a slowdown in the S-phase in HNC mediated by p16ink4a [46], a protein highly expressed in HPV^+^ tumors. In our work, TLR9 was more highly expressed in HPV^+^ OPSCC when compared with HPV^–^ and p16 expression was also higher in HPV^+^ cell lines and tumor specimens (data not shown). Moreover, HPV+ OPSCC show better prognosis which could be influenced by the slowdown of the cell cycle caused by p16ink4a, as demonstrated by these authors.

In cervical cancer, studies have also shown increased expression of TLR9 in those patients who eliminated the virus [15], however this increase in TLR9 may be linked to viral clearance rather than being a characteristic feature of HPV^+^ cervical disease [47].

There are many possible explanations for the poor correlation we have observed between mRNA and protein expression in TLRs 1, 2, 4, 6 and 9 including variations in mRNA half-life [48, 49], and rates of recycling and degradation of proteins. Discrepancies between mRNA and protein expression such as this are not unusual, with only approximately 40% of reported mRNA levels correlating with protein expression [50, 51]. Our study has assessed TLR4 status using flow cytometry, a technique which allows detection of cell surface protein and not endocytosed receptor. TLR4 could be internalized and not detected during the usual cellular processes of storage and recycling [52], and thus membranous TLR4 would not correlate with either mRNA expression or immunohistochemical staining of cytoplasm within tumor specimens. This is demonstrated by the immunohistochemistry stain where is possible to see a very strong stain in the cytoplasm, which may represent internalized TLR4.

TLRs 3, 5, 7 and 8 appear to exert anti-tumor effects by converting immune tolerance into anti-tumor immunity [8]. Our results suggest that TLR3 expression does not correlate with HPV status of cell lines, findings which are consistent with previous studies on TLR3 expression in HPV^+^ OPSCC, *in vivo* [13]. TLR5 also appears to have potent antitumor effects in animal models. The only study of TLR5 in OPSCC demonstrated a decrease in TLR5 in OPSCC p16^+^ [13]. TLR7 in dendritic cells appears to have immunomodulatory and anti-tumor potential [53], but little is known about the contribution of this receptor in carcinogenesis [8]. TLR8 and TLR10 were not expressed in any of the OPSCC lineages studied and further investigations are required.

In summary, we report that of the TLRs, only TLR1 and TLR6 demonstrated reduced mRNA expression in HPV^+^ lineages compared to HPV^-^, and that TLR1, 2, 4, 6 and 9 proteins were expressed in all cell lines, irrespective of HPV-status. TLR9 was upregulated in HPV^+^ OPSCC cell lines, which may represent a cellular response to viral infection. After stimulation with PGN, both HPV^–^ and one HPV^+^ cell lines expressed IL-6 and/or IL-8. We have furthermore demonstrated lower expression of TLR4 in HPV^+^ OPSCC compared to HPV^–^ OPSCC tumor tissue, as well as a lack of IL-6 and IL-8 expression in HPV^+^ cell lines after stimulation with both LPS and LPS-UP, inferring functional changes in the TLR4 signaling pathway of HPV^+^ disease. This work demonstrates a comprehensive survey of TLR expression and function in both HPV^+^ and HPV^–^ OPSCC.

## MATERIALS AND METHODS

Ethical approval was obtained from the School of Dentistry/University of São Paulo Ethical Committee.

### Cell lines and cell cultures

Experiments were carried out using two HPV16-associated oropharyngeal carcinoma cell lines (SCC2 and SCC90) and two HPV negative oropharyngeal carcinoma cell lines (SCC72 and SCC89) (Supplementary Table 1). The cells were received under material transfer agreement from Prof. Susanne M. Gollin, University of Pittsburgh and tested to confirmed HPV status by PCR using HPV 16E1 (Applied Biosystem, UK) in addition to a custom HPV16 E6 probe using previously published sequence of 5′-(FAM)-CCCAGAAAGTTACCA CAGTTATGCACAGAGCT-(TAMRA)-3′ [54]. Short tandem repeat (STR) profiling was undertaken to confirm cell line authenticity. HeLa and THP1 cell lines were used as controls [55] (Supplementary Table 1). Monolayer cultures were grown in Dulbecco’s Modified Eagle’s Medium (DMEM) (Sigma-Aldrich, Dorset, UK) supplemented with 10% FBS and 1 mmol/L L-glutamine and penicillin-streptomycin. THP1 cells were cultured in suspension using RPMI 1640 medium (Sigma-Aldrich, Dorset, UK). Cells were grown in a humidified incubator (5% CO2) at 37^°^C.

### RNA extraction and analysis

RNA was isolated using RNeasy Mini Kit (Qiagen, Manchester, UK) and treated with DNase I (Qiagen, Valencia, CA, USA). The quantity and quality of RNA were analyzed by NanoDrop Spectrophotometry (Thermo Scientific, Hemel Hempstead, UK). Reverse transcription was performed in 300 ng/ml of total RNA using High Capacity cDNA Reverse Transcription (Applied Biosystems, California, USA) in a final volume of 10 mL. Samples were incubated at 25^°^C for 10 min, 37^°^C for 2 h, 85^°^C for 5 min and then kept at 4^°^C using the DNA Engine Dyad thermal cycler.

Supplementary Table 2 summarizes primer sequences used in the experiments. TLR4 and TLR9 primers were based on previously published sequences [22]. IL-6, IL-8 and U6 primers were a gift from Dr. Daniel Lambert (University of Sheffield). Sequence specificity was confirmed using the NCBI-GenBank database and Primer-BLAST. Real-time PCR was performed on cDNA using SYBR green mastermix (Applied Biosystems). Experiments were run in triplicate for 40 cycles at 50^°^C for 2 min, 95^°^C for 10 min, 95^°^C for 15s, 60^°^C for 1 min, using dissociation curve analysis to confirm no bimodal curve or abnormal amplification (Supplementary Figure 1). Fold differences in TLR1-10, IL-6 and IL-8 gene expression were normalized to the housekeeping gene U6. The mean threshold cycle (Ct) reading, from each triplicate experiment, was used to calculate relative gene expression levels. qPCR was performed using an ABI 7900HT PCR machine (Life Technologies, Paisley, UK) and relative mRNA expression calculated using RQ Manager 1.2.1 (Life Technologies, Paisley, UK). Data analysis was then performed using 2^-ΔΔCt.^

For comparative analyses, differences were assessed in terms of TLR expression between cell lines using SCC72 as the calibration sample (random allocation). In the case of TLR2, TLR4, IL-6 and IL-8 expression, differences were assessed between control and stimulated cells (PGN or LPS or LPS-UP) from the same cell line. Ct values exceeding 35 cycles were not considered amplified. Negative control consisted of the master mix, primers and sterile water.

### Flow cytometry

Adherent cells were washed with FACs buffer (PBS, 0.1% sodium azide and 1% BSA) and detached non-enzymatically, centrifuged and re-suspended at 1 × 10^5^ cells/ml in cold FACs buffer on ice. Cells were divided into 4 aliquots and incubated with either PE-conjugated anti-human CD281 (TLR, eBioscience, 12-001141), PE-eFluor 610-conjugated anti human CD282 (TLR2, eBioscience, 61-9922-41), Alexa Fluor700-conjugated anti human CD284 (TLR4 eBioscience, 569917-41) or anti-human CD286 biotinylated (TLR6, eBioscience, 14-9069-80) for 20 min on ice, in the dark, followed by FACS buffer washes. Samples previously incubated with anti-TLR6 biotinylated antibody were incubated for another 20 min with Straptavidin FITC (eBioscience, 11-4317-87). Cells were then washed, centrifuged and fixed with 1% paraformaldehyde. For intracellular staining, cells were fixed in 1% paraformaldehyde, washed with FACS buffer, centrifuged and incubated with 0.1% saponin for 15 min at room temperature and then washed, centrifuged, re-suspended in cold FACS buffer. Cell suspensions were then divided into 2 aliquots and incubated in the dark and on ice with either APC-conjugated anti human CD289 (TLR9, eBioscience, 17-9099-80) or APC-conjugated IgG2a K Isotype control (eBioscience, 17-4321-41). Unstained cells were used as a further control. Experiments were undertaken in triplicate and data is presented as median fluorescence intensity (MFI). Flow cytometry was performed using the LSRII Flow Cytometer System and data analyzed using Flowing Software 2.5.

### Western blotting

Cells were lysed using RIPA buffer (Sigma Aldrich, Poole, UK) containing protease and phosphatase inhibitors (Roche, West Sussex, UK). Samples were centrifuged and the supernatant assayed for total protein using BCA Protein Quantitation as per manufacturer’s protocol (Thermo Scientific, Hemel Hempstead, UK). 20 μg of total protein was loaded onto 4–12% polyacrylamide precast gels (NuPAge Bis-tris mini gels, Novex). After transfer to nitrocellulose membranes using an iBlot Dry Blotting System (Life Technologies, CA, USA) for 7 min, the membranes were washed with Tris buffer and blocked with 5% dried milk in tris buffered saline containing 0,05% tween-20, for 1 h and incubated overnight at 4^°^C with primary monoclonal antibody anti-TLR2 (1:2000, Abcam, ab108998) , or incubated for 1h with primary anti-b-actin (1:3000, Sigma Aldrich, Poole, UK). Membranes were then incubated in horseradish peroxidase-conjugated anti-rabbit IgG (1:3000) for 1 h and developed with SuperSignal West Pico chemiluminescent substrate (Thermo Scientific, Hemel Hempstead, UK). THP1 lysate was used as a positive control.

### Stimulation of OPSCC cell lines with LPS, LPS-UP and PGN

SCC24, SCC90, SCC72, SCC89 were stimulated with various concentrations of lipopolysaccharide (LPS: a non-specific TLR4 agonist) from *Escherichia coli* 0111:B4 (Sigma-Aldrich, St. Louis, MO, USA), lipopolysaccharide ultra-pure (LPS-UP: a specific TLR4 agonist) from *E. coli* (*In vivo* Gen, San Diego, California, USA), and peptidoglycan from *Staphylococcus aureus* (PGN: a TLR2 agonist) (Sigma-Aldrich, St. Louis, MO, USA). For ELISA, cells were stimulated with 0.01–1 µg/mL of LPS; 0.01-1 µg/mL of LPS-UP and 0.1–10 µg/mL of PGN [25, 56]. For qPCR and Western blot, cells were stimulated with 1 µg/mL of LPS or LPS-UP or PGN. All incubations were undertaken in a humidified incubator (5% CO2) at 37^°^C for 24 h. Experiments were undertaken using triplicate biological repeats.

### Measurement of cytokine levels

LPS, LPS-UP and PGN were added to keratinocyte monolayers (SCC2, SCC90, SCC72 and SCC90) in T25 flasks. Culture supernatants were collected in order to measure cytokine levels of IL-6 and IL-8, using BD OptEIA Human IL-6 and BD OptEIA Human IL-8 ELISA kits (BD Biosciences, Torreyana Road, San Diego, CA, USA).

### Analysis of TLR4 expression in OPSCC tissue

We analyzed TLR4 expression in 61 FFPE oropharyngeal tumor samples in a tissue microarray (TMA) consisting of 31 HPV^+^ oropharyngeal carcinomas (Supplementary Table 3) and 30 HPV^–^ carcinomas (Supplementary Table 4) (from Prof. Mark Lingen, University of Chicago). Two 1 mm cores, taken from the body of each tumor were available for each case. The TMA slide was incubated overnight at 37^°^C, then deparaffinized and hydrated and washed with Tris buffer. This was followed by antigen retrieval in 10 mM sodium citrate (pH 6.0) at 95^°^C for 30 mins, and quenching in 3% hydrogen peroxide for 15 min. Blocking of nonspecific binding was undertaken using 5% BSA at room temperature for 30 mins. The tissue was then incubated overnight at 4^°^C with Monoclonal Anti-TLR4 (1:1000, Abcam, ab22048), followed by the Envision Dual Link System horseradish peroxidase method. Staining was then revealed by the addition of di-aminobenzidine (DAB) substrate-chromogen and the TMA was counterstained, dehydrated and mounted.

### Semi-automated quantification of histochemical staining by color deconvolution

Digital image of IHC-stained TMA slide was obtained using a digital slide scanner (ScanScope-Aperio). Tumor area was then calculated using ImageJ software, by selecting the region of interest using the “measure” tool. Each spot image was submitted to color deconvolution [57] to separate the blue color from hematoxylin and the brown color from DAB using the plugin in ImageJ software (National Institute of Health). The positive labeling (brown color) was selected using the threshold tool of ImageJ (from 0 to 127 brown tones). The final score was calculated as [(positive labeling area/tumor area) ×100)].

### Statistical analysis

Statistical analysis was undertaken using GraphPad Prism software version 6.0 (GraphPad Prism Software, San Diego, CA, USA). For qPCR data, results are expressed as mean ± SEM, and statistical significance assessed using one-way ANOVA post hoc Tukey analysis to compare more than 2 groups and t-Student to compare two independent groups. For flow cytometry data, after failure of normality testing, the non-parametric Kruskal-Wallis test with post hoc Dunn-Bonferroni correction was used. Graphs depict mean MFI ± SD. Analysis of ELISA data was undertaken through comparison of stimulated samples (LPS, LPS-UP or PGN) against unstimulated control, using the Mann-Whitney *U*-test. Graphs are expressed as pg/mL and mean ± SD. The Mann-Whitney U test was also used to compare TLR4 staining between HPV^+^ and HPV^–^ tumors. All statistical tests were undertaken with the significance level set at *p <* 0.05.

## SUPPLEMENTARY MATERIALS FIGURES AND TABLES



## Abbreviations

(TLRs): Toll-like receptors
(HPV): human papillomavirus
(OPSCC): Oropharyngeal Squamous Cell Carcinoma
(qPCR): real-time polymerase chain reaction
(LPS): lipopolysaccharide
(LPS-UP): LPS ultra-pure
(PGN): peptidoglycan
(IL): interleukin
(PAMP): pathogen-associated molecular patterns
(PRRs): pattern-recognition receptors
(mRNA): ribonucleic acid
(Rb): retinoblastoma
(NOD): nucleotide-binding oligomerization domain
