# Supplementary material for: Altered Toll-like receptor expression and function in HPV-associated oropharyngeal carcinoma
Source: Oncotarget. 2017 Jul 4;9(1):236–48. doi: 10.18632/oncotarget.18959 (PMC5787461; doi:10.18632/oncotarget.18959)
Supplement: Supplementary file 1 [file oncotarget-09-236-s001.pdf]

Altered Toll-like receptor expression and function in HPV-associated oropharyngeal carcinoma

SUPPLEMENTARY MATERIALS

Supplementary Table 1: Cell lines used in this study

Cell line	TNM	Site	Sex	HPV
UD-SCC2	T1N2	Hypopharynx	Male	HPV16
UPCI-SCC90	T2N1	Base of tongue	Male	HPV16
UPCI-SCC72	T3N2	Tonsil	Female	HPV-
SCC89	T4N2	Tonsil	Male	HPV-
HELA		Uterine Cervix	Female	HPV18
THP1	N/A	Monocyte	Not known	HPV-

Supplementary Table 2: List of sequences of forward and reverse primers

Gene	Foward 5′– 3′	Reverse 5′–3′	Product size	Genbank
TLR1	CTGTGAACCTCAAGCACTTGG	GAGCAATTGGCAGCACACTA	144	NM_003263.3
TLR2	TAACAGGCTGCATTCCCAAGA	TAGTAACATGGGTAAGAGGGAGG	155	NM_001318796.1
TLR3	GTGGATAGCTCTCCTTCACCA	CAGAGCCGTGCTAAGTTGTTA	155	NM_003265.2
TLR4	AGTTTCCTGCAATGGATCAAGG	CTGCTTATCTGAAGGTGTTGCAC	84	NM_138554.4
TLR5	GATTCTTGCCCACCACATCAT	CAGGTGTCTCACTGAACTTCTG	106	NM_003268.5
TLR6	GATATCCTGCCATCCTATTGTGAG	CAGCTTCATAGCACTCAATCCC	133	NM_006068.4
TLR7	TGGAAATTGCCCTCGTTGTT	GTCAGCGCATCAAAAGCATT	99	NM_016562.3
TLR8	CAGCCTGGGAAAGGAGACTA	TCTTCGGCGCATAACTCACA	105	NM_016610.3
TLR9	AGTCAATGGCTCCCAGTTCCT	CGTGAATGAGTGCTCGTGGTA	94	NM_017442.3
TLR10	TCCAGAATGAGTGGTGCCAT	CCTTGGGCCATTCCAAGTATG	177	NM_030956.3
IL-6	AATAAAACAACCTGAACCTTCCA	TTATTGATTTTCACCAGGCAAGT	94	NM_001318095.1
IL-8	TGTGTGAAATTATTGTAAAGCTT	TAAATCTTCTCCACAACCCTCTG	91	NM_000584.3
U6	CTCGCTTCGGCAGCACA	AACGCTTCACGAATTTGCGT	96	NM_020810.3

Supplementary Table 3: HPV-positive tumor species data

	Sex	Age at the diagnosis	Race	Site	TNM	Smoke (Years of use)	Drink	Recurrence	Metastasis	Survival (Months)
1	M	50	White	Left Tonsil	T2N0	N	N	N	N	59
2	M	58	White	Left tonsil	T2N2b	N	N	N	N	30
3	M	66	White	Left tonsil	T2N2aM0	N	N	N	N	37
4	M	66	White	Right tonsil	T4aN1	N	N	N	N	55
5	M	45	White	Oropharynx	TxN2b	N	N	N	N	24
6	M	65	White	Right Tonsil	T3N2b	N	N/I	N	N	3
7	M	55	White	Left tonsil	T3N3	Former (5)	N	N	N	23
8	F	52	White	Based of tongue	T1N2c	Former (3)	N	N	N	32
9	M	61	White	Left Tonsil	T4bN2b	Former (11)	N	N	N	9
10	M	67	White	Right tonsil	T2N2b	Y (30)	Y	N	N	70
11	M	70	White	Tonsil	T2N2bM0	Former (25)	N	N	N	7
12	M	58	White	Left tonsil	T1N1	Former	N	N	N	65
13	M	54	Black	Right Tonsil	T4N2b	Former (45)	N	N	N	63
14	M	81	White	Left tonsil	T2N0	Former (30)	N	N	N	23
15	M	70	N/i	Right Tonsil	T2N2b	Y	N	N	N	Deceased *
16	M	53	White	Left tonsil	T3N2bM0	N	N	N	N	65
17	M	74	White	Right tonsil	T3N2b	Y	Y	N	N	Deceased *
18	F	42	White	Left tonsil	T4N2	Y	Y	N	N	N/I
19	M	67	Black	Right tonsil	T2N0	N	N	N/I	N/I	Deceased
20	M	47	White	Neck	N/I	Former	N	N	N	41
21	M	45	White	Right Tonsil	T2N0	N	N	N	N	52
22	M	72	White	Right Tonsil	T2N2b	Former (30)	N	N	N	39
23	M	55	White	Right Tonsil	T2N2a	Y (5)	N	N	N	5
24	F	66	White	Left tonsil	T1aNx	Former	N	N	N	29
25	M	65	White	Left Tonsil	T2Nx	Y (40)	N	N	N	33
26	M	53	White	Left Tonsil	T1N1	Former	N	N	N	33
27	F	91	White	Based of tongue	T1Nx	N	N	N	N	N/I
28	M	63	White	Based of tongue	N/I	N	N	N	N	N/I
29	M	66	White	Based of tongue	T2N0	N	N	N	N	18
30	M	54	White	Neck	T1N2	N	N	N	N	22
31	M	65	White	Based of tongue	T3N2cM0	Former (50)	N	N	N	26

M = Male; F = Female; N/I = not informed; Y = Yes; N = No; *Unknown cause, Survival in months since diagnosis.

Supplementary Table 4: HPV-negative tumor species data

	Sex	Age at the diagnosis	Race	Site	TNM	Smoke (Years of use)	Drink	Recurrence	Metastasis	Survival (Months)
1	F	62	White	Floor of mouth	T1N1	Y (40)	Y	N	N	N/I
2	F	52	White	Tongue	T2N2c	Y (30)	Y	N	N	60
3	M	68	White	Tongue	T3N1	Y (50)	Y	Y	N	Deceased
4	M	71	Black	Larynx	T4aN2b	Y (40)	N/I	Y	N	Deceased
5	M	68	White	Tongue	T3Nx	Y (42)	Y	N	N	N/I
6	M	69	White	Pharynx	T1Nx	Y (60)	N/I	Y	N	55
7	M	63	White	Pharynx	T4aN1M1	Y (40)	N	N	N	N/I
8	M	53	White	Soft palate	T2N2bM0	Former	Y	N	Neck	N/I
9	F	62	White	Tongue	T2N1	Y (20)	N/I	Y	N	N/I
10	M	58	N/I	Hypopharynx	T2Nx	Y	Y	Y	N	59
11	F	59	White	Tongue	T2N0Mx	N	N	N	N	59
12	M	70	White	Larynx	T4N0	Former (15)	Y	N	N	Deceased
13	F	65	N/I	Tongue	T2N1	N	N	N	N	N/I
14	M	62	White	Tongue	T2N0	Former (4)	N	N	N	48
15	F	62	White	Palate	T4aN0	Former (30)	Y	Y	Lung	N/I
16	M	26	White	Tongue	T2N2b	Y (15)	N	N	N	60
17	M	54	White	Right cheek	T2N2b	Former (15)	N	N	N	69
18	M	53	White	Tongue	T4N0	Y (37)	Y	Y	N	N/I
19	M	57	White	Tongue	T2N0	Y (38)	Y	N	N	46
20	M	70	Black	Tongue	T4aN2b	Y	Y	Y	Lung	Deceased
21	M	53	White	Palate	T3N0	Former (20)	N	N	N	45
22	M	75	White	Tongue	T2N2b	N	N	N	Bone	Deceased
23	F	80	White	Tongue	T2N2	Former	N	Y	N	Deceased
24	M	69	White	Floor of mouth	T2N0	Former (40)	N	Y	N	47
25	M	49	White	Tongue	T4a Nx	Y (42)	N	Y	Neck	N/I
26	F	74	White	Tongue	T2N2b	N	Y	N	N	N/I
27	M	56	White	Larynx	T2N2	Y (40)	Y	Y	Lung	N/I
28	F	65	White	Buccal	T1N0	Former (40)	Y	Y	N	1
29	M	72	White	Larynx	T3N0	Former (45)	N	N	N	7
30	M	58	White	Tongue	T1N1	Former	N	N	Neck	20

M = Male; F = Female; N/I = not informed; Y = Yes; N = No; Survival in months since diagnosis.

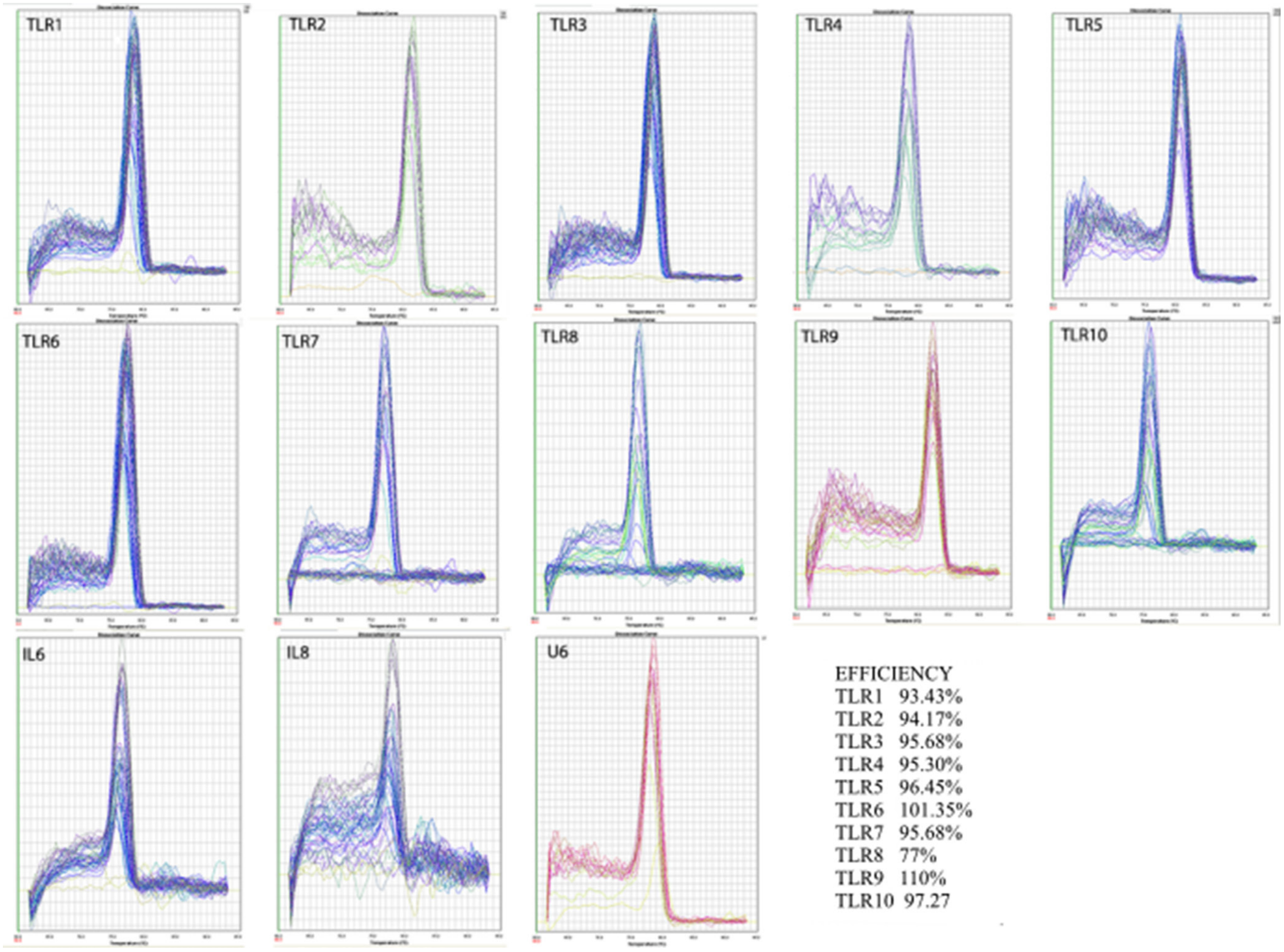

Supplementary Figure 1: Melting curve.

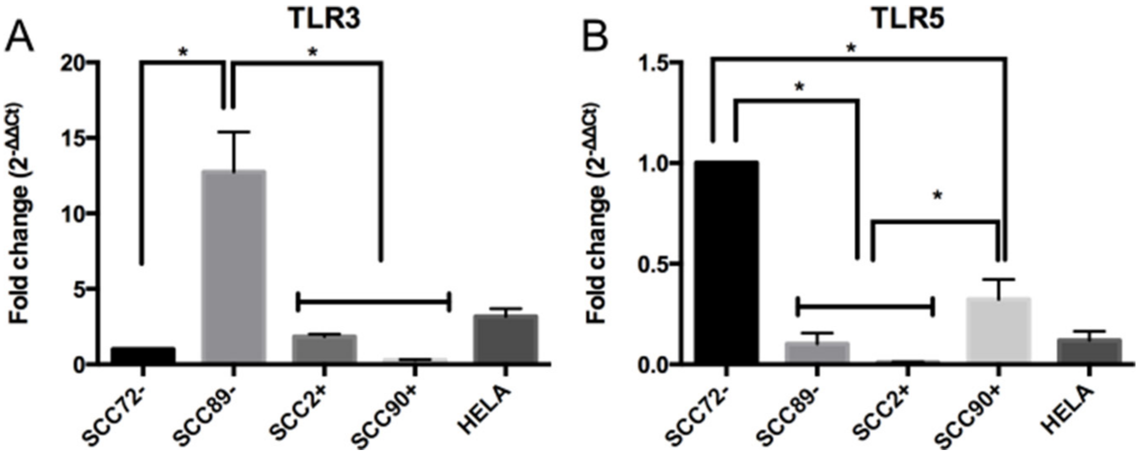

Supplementary Figure 2: (**A**) TLR3 was significant higher expressed in SCC89 (HPV^-) compared to the other cell lines (normalized to U6). (**B**) TLR5 was significant lower in SCC89 (HPV^-), SCC2 (HPV^+) and SCC90 (HPV^+) compared to SCC72 (HPV^-), and SCC90 was significant higher than SCC89 and SCC2 (normalized to U6). ($^*p < 0.0001$; mean ± SEM).
